# PLOD2, a key factor for MRL MSC metabolism and chondroprotective properties

**DOI:** 10.1186/s13287-024-03650-2

**Published:** 2024-03-07

**Authors:** Sarah Bahraoui, Gautier Tejedor, Anne-Laure Mausset-Bonnefont, François Autelitano, Audrey Barthelaix, Claudia Terraza-Aguirre, Vincent Gisbert, Yoan Arribat, Christian Jorgensen, Mingxing Wei, Farida Djouad

**Affiliations:** 1 IRMB, University of Montpellier, INSERM U 1183, Hôpital Saint-Eloi, 80 Avenue Augustin Fliche, 34295 Montpellier cedex 5, France; 2 EVOTEC (France) SAS, 31100 Toulouse, France; 3 Clinical Immunology and Osteoarticular Disease Therapeutic Unit, Department of Rheumatology, CHU Montpellier, 34095 Montpellier, France; 4 CellVax, Villejuif Bio Park, 1 Mail du Professeur Georges Mathé, 94800 Villejuif, France

**Keywords:** MRL mouse, Regeneration, Mesenchymal stem cells, PLOD2, Metabolism, Chondroprotection

## Abstract

**Background:**

Initially discovered for its ability to regenerate ear holes, the Murphy Roth Large (MRL) mouse has been the subject of multiple research studies aimed at evaluating its ability to regenerate other body tissues and at deciphering the mechanisms underlying it. These enhanced abilities to regenerate, retained during adulthood, protect the MRL mouse from degenerative diseases such as osteoarthritis (OA). Here, we hypothesized that mesenchymal stromal/stem cells (MSC) derived from the regenerative MRL mouse could be involved in their regenerative potential through the release of pro-regenerative mediators.

**Method:**

To address this hypothesis, we compared the secretome of MRL and BL6 MSC and identified several candidate molecules expressed at significantly higher levels by MRL MSC than by BL6 MSC. We selected one candidate, *Plod2*, and performed functional in vitro assays to evaluate its role on MRL MSC properties including metabolic profile, migration, and chondroprotective effects. To assess its contribution to MRL protection against OA, we used an experimental model for osteoarthritis induced by collagenase (CiOA).

**Results:**

Among the candidate molecules highly expressed by MRL MSC, we focused our attention on procollagen-lysine,2-oxoglutarate 5-dioxygenase 2 (PLOD2). *Plod2* silencing induced a decrease in the glycolytic function of MRL MSC, resulting in the alteration of their migratory and chondroprotective abilities in vitro. In vivo, we showed that *Plod2* silencing in MRL MSC significantly impaired their capacity to protect mouse from developing OA.

**Conclusion:**

Our results demonstrate that the chondroprotective and therapeutic properties of MRL MSC in the CiOA experimental model are in part mediated by PLOD2.

**Supplementary Information:**

The online version contains supplementary material available at 10.1186/s13287-024-03650-2.

## Introduction

Regeneration ability is a property that varies widely during development and among species. Indeed, while able to regenerate at early embryonic stages, adult mammals will trigger a tissue repair mechanism resulting in scar formation after tissue injury and limiting the structural and functional recovery [1, 2]. In contrast, some species, such as salamander [3], zebrafish [4] and hydra [5, 6], maintain their regenerative properties during their all-life [7–9]. Rare exceptions, such as the super healer Murphy Roths Large (MRL) mouse, exist among mammals. Indeed, the adult MRL mouse is a competent model for tissue regeneration, suggesting that crucial regenerative mechanisms are conserved in this mammalian model. MRL mouse possesses the extraordinary potential to regenerate multiple musculoskeletal tissues such as the outer ear, articular cartilage, and digits without scarring [10–15]. Among the conserved mechanisms underlying regeneration, the emphasis on aerobic glycolytic energy metabolism has been reported to be essential in several regenerative species including the MRL mouse [16–18].

MRL mice are protected from developing joint diseases such as osteoarthritis (OA) [11, 19]. Identifying the mechanisms underlying articular cartilage regeneration and protection from osteoarthritis (OA) would allow the development of novel therapies for OA patients. Indeed, OA is a complex disease characterized partly by the degradation of articular cartilage and for which no curative treatment exists to date. One therapeutic option studied is the intra-articular administration of mesenchymal stem/stromal cells (MSC). The trophic activities of MSC introduced exogenously have been shown to protect from cartilage degradation and OA development in the collagenase-induced OA (CiOA) model [20–22]. This experimental model reproduces some events characteristic of the human OA disease, such as moderate inflammation of the synovial membrane and, the destruction of the cartilage [23, 24]. Indeed, such as resident, exogenous MSC participate in joint homeostasis through a paracrine action that contributes to repairing damaged tissue, restoring tissue metabolism, and preventing inflammation [22, 25].

In the context of digit tip regeneration in mice, mesenchymal cells from one type of tissue were shown to participate in the regeneration of other mesenchymal tissues [26]. The authors showed that the regenerative environment primes the cells from injured tissues to acquire a blastema mesenchymal transcriptional state enabling them to regenerate other mesenchymal tissues such as the dermis. Therefore, it can be speculated that identifying regenerative environmental factors could stimulate the regenerative potential of MSC in adult mammals. The use of adult MSC in experimental models of degenerative diseases such as OA have shown promising results [20, 27, 28]. However, while MSC prevent cartilage degradation and OA development when administrated locally their capacity to regenerate damaged OA cartilage has never been proven.

The cartilage regenerative potential of MRL mice and their subsequent resistance to experimental osteoarticular defects has increased the interest in MSC derived from MRL mice (MRL MSC). Therefore, we hypothesized that MRL MSC regenerative and protective properties might be associated with their intrinsic properties, particularly their aerobic glycolytic energy metabolism controlled through soluble factors. To address that hypothesis and identify soluble factors presumably at the origin of MRL mouse resistance to OA, we performed a comparative study of MRL MSC and BL6 MSC secretome. Considering the glycolytic metabolic profile of the MRL mouse, we investigated the role of one of the genes overexpressed by MRL MSC: *Plod2*, in their regenerative capacities.

## Materials and methods

### MSC isolation and expansion

Mesenchymal Stem/Stromal Cells (MSC) from MRL/Mpj (MRL MSC) and C57BL/6 (BL6 -MSC) mice bone marrow were isolated and expanded as previously described [29].

### Murine chondrocyte culture and co-culture

Murine articular chondrocytes were isolated from the knees and femoral head of 3-day-old C57BL/6 mice as described previously [30, 31]. Briefly, chondrocytes (25 000 cells/cm^2^) were plated in 12-well culture plates (TPP Techno Plastic Products, Switzerland) with 1 mL of proliferative medium for five days. Then, chondrocytes were treated with 1 ng/mL Il-1β (R&D Systems) for 24 h (day 0) to generate the so-called "OA-like" chondrocytes. For co-culture experiments, 2 × 10^5^ of naïve or modified MSC were seeded in 12-well culture inserts with 1 mL of proliferative media and co-cultured with OA-like chondrocyte for 24 h (day 1). 48 h later, chondrocytes were recovered (day 3) and processed for RT-qPCR.

### MSC transfection with siRNA for PLOD2 silencing

MRL MSC were grown in 6-well plates until subconfluence (70%), then transfected overnight with 50 nM of control siRNA (siCTL) or the siRNA against PLOD2 (siPLOD2) (Silencer® pre-designed siRNA, Ambion, Life Technologies™; 5’-GCU AUG GAG CAC UAC GCC A dTdT-3) using 6 µl of Oligofectamine reagent per well (Life Technologies, Courtaboeuf), according to the supplier's recommendations. MRL MSC were used for follow-up experiments at 48 h post-transfection.

### MSC transfection with CMV plasmid for PLOD2 overexpression

MSC derived from BL6 mice were grown in 6-well plates until subconfluence (70%) and transfected with 5 µg of PLOD2 plasmid (CMV PLOD2) (pRP[Exp] -mCherry-CMV > mPLOD2 [NM_001142916.1], Vector Builder) or Control (pcDNA-Cherry) using Lipofectamine 3000 reagent (Life Technologies, Courtaboeuf) following to the supplier's recommendations. Transfection solutions were added to cell medium for 6 h before to wash. Transfection level was confirmed after 48 h by using fluorescent microscope (ThermoFisher EVOS™ M5000 Imaging System) prior to perform follow-up experiments.

### RT-qPCR

Total RNA was isolated from mMSC or chondrocytes using the RNeasy Mini Kit (Qiagen, Courtaboeuf), and the quantity and purity of the total RNA were determined using a NanoDrop ND-1000 spectrophotometer (NanoDrop ND, Thermo Fisher Scientific). cDNA was synthesized by reverse transcribing 500 ng of RNA into cDNA using the SensiFAST™ cDNA Synthesis Kit (Bioline, Meridian Life Science© Company). Quantitative PCR was performed on 6.25 ng of cDNA using the SensiFAST™ SYBR® No-ROX kit (Bioline, Meridian Life Science© Company) and a LightCycler® 480 Detection system (Roche), following manufacturer's recommendations. Specific primers for mouse *Plod2, Cspg4, Inhbb, Efemp1, Lama4, Mmp3, Htra1, Nt5e, Lcn2, C1qtnf5, Fam20c* and *Hif-1α* were designed using the Primer3 software and can be provided upon reasonable request. Primers for *Col2b, Agn, Mmp13, Adamts5* are the same as previously described [31]. Values were expressed as relative mRNA level of specific gene expression as obtained using the 2^−ΔCt^ method, using the *Rsp9* and *ActB* expression as housekeeping genes.

### Western Blot

MSC were lysed in RIPA buffer containing protases inhibitors.

A micro-BCA dosage (ThermoFisher) was used according to the manufacturer indications to load 30ug of protein per conditions. Proteins from whole lysate were separated in Laemmli Buffer by SD-Page and transferred on Nitro-cellulose membrane using iBlot™ 2 Dry Blotting System from Invitrogen. Membranes were blocked in 5% milk in Tris-buffered saline with 0.1% Tween-20 (TBST) and incubated with primary antibodies: 1/1000e of rabbit anti- PLOD2 (proteintech, 21214-1-AP) and 1/2500e of mouse anti-actin (sigma, A5441) overnight at 4 °C. Membranes were washed with TBST, incubated with HRP-conjugated secondary antibodies for 1 h, incubated with HRP substrate, and imaged using a ChemiDoc MP Imaging System (BioRad). Quantification was done by using Image J software.

### Cell viability assay

We assessed the proliferation rate of the cells using the CellTiter-Glo® Luminescent Cell Viability Assay from Promega. Briefly, 1300 cells/well of a 96-well plate were seeded in triplicate, and the luminescence signal at 0 h, 24, 48 and 72 h was measured according to the manufacturer instruction.

### Scratch wound healing

Migratory potential of the cells was assessed with scratch wound healing assay. 2.5 × 10^5^ cells were seeded in TC24 plates and maintained at 37 °C with 5% of CO2 in proliferating media. The wound was performed manually once the cells adhered to the plastic and reached 90% confluency. Wound closure was studied using an inverted microscope (EVOS M5000, Thermo Fisher Scientific), and images of the scratch were taken at H0, just after the scratch and at H24 to evaluate the wound closure. The wounded area was measured at H0 and H24 using Image J Software: the open wound area (in percentage) was calculated by comparing H0 and H24 images and normalized to H0.

### Real-time cellular metabolic flux assays

Oxygen consumption rate (OCR) and extracellular acidification rate (ECAR) were measured using the XF96 analyzer (Seahorse Biosciences, North Billerica, MA, USA). Transfected and non-transfected murine MSC were plated on 96-well plates 6 h before the experiment in XF media (non-buffered DMEM medium, containing 5 mM glucose, 2 mM L-glutamine, and 1 mM sodium pyruvate. OCR and ECAR were measured under basal conditions and in response to 25 mM D-Glucose, 2 μM of oligomycin, 2 μM of carbonylcyanide-4-(trifluoromethoxy)-phenylhydrazone (FCCP) and 0.5 μM of antimycin A and rotenone (Seahorse XF Cell Energy Phenotype Test Kit from Agilent).). Three successive readings were taken after each sequential injection. The instrumental background was measured in separate control wells using the same conditions without biologic material. After the seahorse experiment, the plated cells were fixed for 10 min in 4% PFA and then, stained with HOESCHT for 5 min (1/8000e) to count cells with Agilent BioTek Cytation for normalization.

### L-Lactate quantification

Following 48 h after transfection, cells were cultured in DMEM without phenol red, supplemented with 10% FBS (Biowest, Nuaillé, France), 25 mM D-glucose (ThermoFisher Scientific, Whaltham, MA, USA), 2 mM L-glutamine and 1 mM sodium pyruvate (Gibco – ThermoFisher Scientific). Conditioned medium was recovered after 24 h and centrifuged at 400×*g* for 10 min at 4 °C to remove cell debris. Lactate was quantified using L-Lactate Assay Kit colorimetric (Abcam, Cambridge, UK) according to the provider’s instructions. For this purpose, several dilutions of the samples were prepared in Lactate assay buffer and the absorbance was measured at OD 450 nm in a microplate reader. Cell number for each condition was used for data normalization.

### Collagenase-induced osteoarthritis (CiOA) mouse model and histological analysis

Mice used for this study were housed and cared in accordance with the European directive 2010/63/EU. The CiOA model was generated upon the approval from the Ethical Committee for animal experimentation of the Languedoc-Roussillon and the French Ministry for Higher Education and Research (Approval #5349-2016050918198875 v3). Briefly, 1U type VII collagenase in 5 μL saline was administrated in the intra-articular (IA) space of C57BL/6 mice knee joints (10 weeks old) at day 0 and 2. Groups of 10 mice received MSC (2.5 × 105 cells/5 μL saline) at day 7. At day 42, mice were euthanatized by exposure to CO_2_ until complete cessation of breathing was observed followed by cervical dislocation, and paws were recovered for fixation in 4% formaldehyde and decalcified in 4% EDTA solution for three weeks before paraffin embedding. Tibias were sectioned frontally as previously described [20, 31, 32] and stained with safranin O fast green. Two persons performed blind quantification of the degradation of cartilage using the modified Pritzker OARSI score as described [20, 31, 32]. Mice corresponding to uninterpretable stained slides were removed from the analysis.

### Statistical analysis

All data are presented as the mean ± Standard Error of the Mean (SEM), and all experiments were performed at least three times. The Student's *t* test was used to compare two experimental groups, and ANOVA followed by a Friedman test for multiple comparison of paired samples was used for the co-culture experiments while ANOVA with Kruskal–Wallis test for multiple comparisons of non-paired samples was used for the CiOA. Graphs show mean ± Standard SEM. *P* values < 0.05 (*), *P* < 0.01 (**) or *P* < 0.001 (***) were considered statistically significant. Analysis and graphical representation were performed using Graph-Pad Prism™ software (Graphpad).

## Results

### MRL MSC exhibit a specific secretome as compared to MSC derived from C57BL/6 mice

**Fig. 1:**
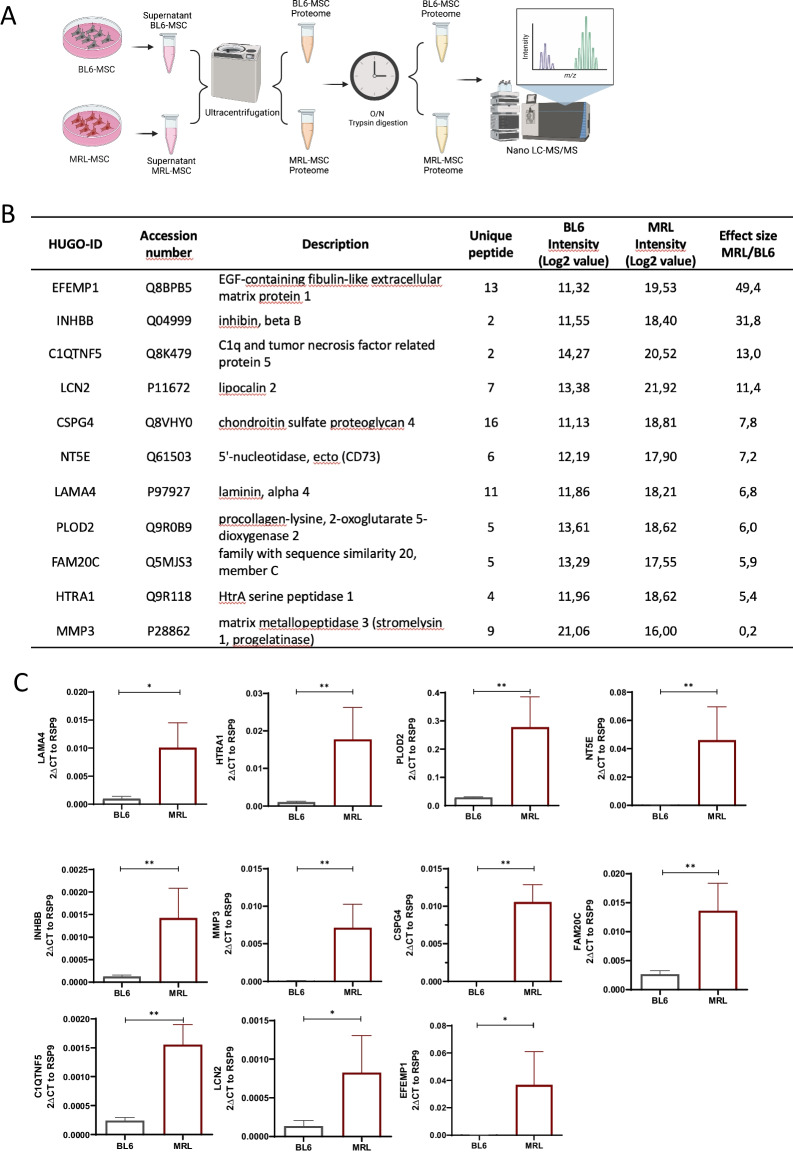
Secretome and expression profiles of MRL MSC and BL6 MSC. **A** and **B** Proteomic analysis of differential expression of *Plod2, Inhbb, Efemp1, Lama4, Mmp3, Htra1, Nt5e, Lcn2, C1qtnf5, Fam20c* in MRL MSC compared to BL6 MSC. “Effect size” indicates the standardized mean difference in protein expression level between MRL MSC and BL6 MSC. The median intensity levels (Log 2 value) in MRL MSC and BL6 MSC are indicated for each protein. Normalized protein intensities were used to calculate the Effect size MRL/BL6. **C** *Plod2, Inhbb, Efemp1, Lama4, Mmp3, Htra1, Nt5e, Lcn2, C1qtnf5, Fam20c* expression levels in MRL MSC compared to in MRL MSC compared to BL6 MSC. Error bars represent mean ± SEM. *P < 0.05; **P < 0.01; ***P < 0.001, (*n* = 6)

We recently performed label-free quantitative shotgun proteomics to identify differentially secreted proteins between MRL MSC and BL6 MSC [32]. This published secretome was analyzed *denovo* on the basis of protein intensities quantified by LC–MS/MS with the aim to identify key factors for MRL MSC metabolism and chondroprotective properties (Fig. 1A). Among the 810 proteins differentially expressed between MRL MSC and BL6 MSC by at least 1.5‐fold, 625 proteins were secreted at higher levels by MRL MSC. We focused our attention on proteins with a higher secretory profile by MRL MSC in particular *LAMA4, HTRA1, PLOD2, INHBB, MMP3, CSPG4, NT5E, LCN2, EFEMP1, FAM20C and C1QTNF5* (Fig. 1B). By RT-qPCR, we confirmed that these 11 factors were overexpressed at a significantly higher level in MRL MSC as compared to BL6 MSC (Fig. 1C).

Among the list of 11 factors, we focused our attention on *PLOD2* (procollagen-lysine,2-oxoglutarate 5-dioxygenase 2) overexpressed at protein level by MRL MSC compared to BL6 MSC (Additional file 1: Fig. S1A and S1B). *PLOD2* is known to be regulated by Hypoxia Inducible Factor 1 Subunit Alpha (HIF-1a) [33] a key factor for the regeneration process of adult MRL mice [34]. Moreover, *PLOD2* codes for lysyl hydroxylase LH2 in charge of post-translational modifications of collagen type I for its stability and stiffness [35, 36].

### *Plod2* is required for MRL MSC glycolytic metabolism

**Fig. 2:**
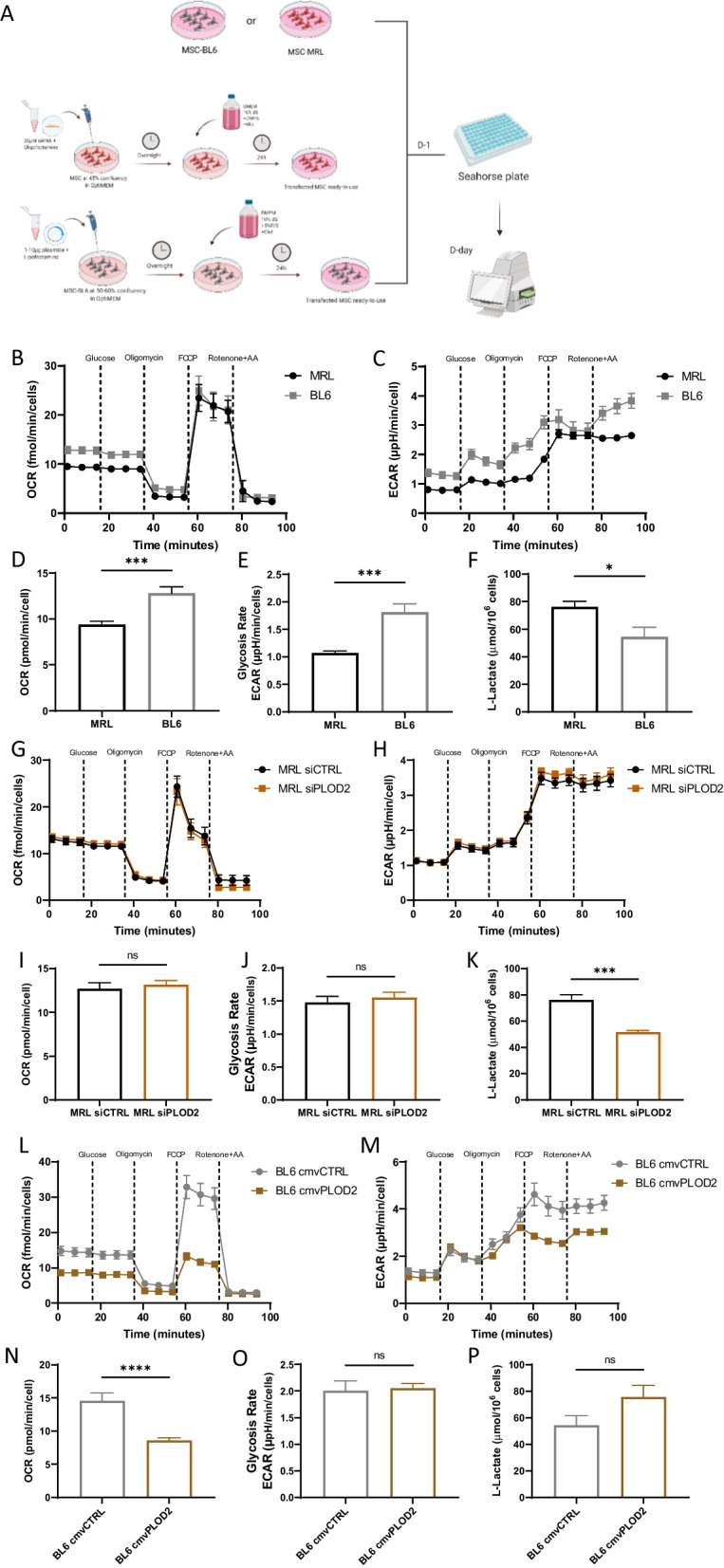
PLOD2 contributes to the specificity of MRL MSC metabolism. Analysis of OCR and ECAR was performed using Seahorse XF analyzer to assess mitochondrial respiration and glycolysis. **A** Schematic workflow illustrates the experimental procedure. Seahorse respirometry assays were performed on control BL6 MSC and MRL MSC, comparing untreated or transfected cells with PLOD2 siRNA and PLOD2 plasmid. **B** and **D** OCR was compared between BL6 MSC and MRL MSC**, G** and** I** between MRL MSC_siCTL_ and MRL MSC_siPLOD2,_** L** and** N** between BL6 MSC and BL6 MSC_+cmv PLOD2_, with sequential addition of D-Glucose 25 mM, oligomycin (Oligo, complex V inhibitor), FCCP (protonophore), and antimycin A (complex III inhibitor)/rotenone (complex I inhibitor) to analyze ATP-linked respiration, basal respiration, maximal respiratory capacity and spare respiratory capacity. **B, G** and **L** represent the global OCR profiles. **D**,** I** and **N** illustrate baseline OCR. **C** and **E** ECAR was compared between BL6 MSC and MRL MSC, **H** and **J** between MRL MSC_siCTL_ and MRL MSC_siPLOD2,_** M** and **O** between MSC BL6 and MSC BL6_+CM PLOD2_ with serial addition of glucose and oligomycin to measure basal glycolysis, glycolytic reserve, maximal glycolysis. **C, H,** and **M** represent the global ECAR profiles. **E**, **J** and **O** illustrate ECAR after acute injection of glucose. **F**, **K** and **P** show L-Lactate concentration in the culture media harvested after 24 h of culture. The concentration was measured using a colorimetric L-Lactate Assay Kit (*n* = 4). **D, E, I, J, N** and **O** all the bar values represent means ± SEM of 5 to 10 technical replicates. *P < 0.05; **P < 0.01; ***P < 0.001, Mann–Whitney unpaired *t* test, two-tailed

MRL mouse uses aerobic glycolysis as their basal metabolic state [37, 38]. First, we wondered whether MRL MSC exhibit a different metabolism than BL6 MSC. To address that question, we compared the oxygen consumption rate (OCR) and the extracellular acidification rate (ECAR) of the two types of MSC by assessing metabolic profile with the Seahorse XF technology (Fig. 2A). We measured lower OCR and ECAR in MRL MSC compared with BL6 MSC (Fig. 2B–E) while lactate production appeared higher (Fig. 2F). Hence, to investigate the role of *Plod2* in this specific profile, we used the small interfering RNA (siRNA) approach to knock down the expression of *Plod2* in MRL MSC. 48 h post-transfection of MSC with a siRNA against *Plod2* (siPLOD2), *Plod2* expression was reduced by 71% compared with the MSC transfected with the control siRNA (siCTL) (Additional file 1: Fig. S1B and S1C). *Plod2* knock down did not significantly alter the proliferation rate of MRL MSC (Additional file 1: Fig. S1D). Then, we quantified the OCR, in MRL MSC transfected with siCTL (MRL) or siPLOD2 (MRL siPLOD2) and found that *Plod2* silencing did not impact OCR and ECAR in MRL MSC (Fig. 2G–J). However, depletion of *Plod2* dramatically reduced lactate production in MRL MSC (Fig. 2K).

Conversely, we asked whether *Plod2* overexpression in BL6 MSC would further enhance their glycolytic activity. To address that question, BL6 MSC were transfected with a plasmid expression murine *Plod2* (BL6 + CMV_PLOD2_) or an empty vector as control (BL6) (Additional file 1: Fig. S1E and S1F). We evaluated the OCR or the ECAR of the cells with Seahorse analyzer and revealed that *Plod2* overexpression in BL6 MSC reduced OCR (Fig. 2L- and N), reaching the same value than MRL MSC (Additional file 1: Fig. S2). ECAR and lactate production were not affected by *Plod2* overexpression (Fig. 2M, O and P). Altogether, these results revealed the role of *Plod2* in MSC metabolic adaptations.

### *Plod2* is required for MRL MSC migration potential

**Fig. 3:**
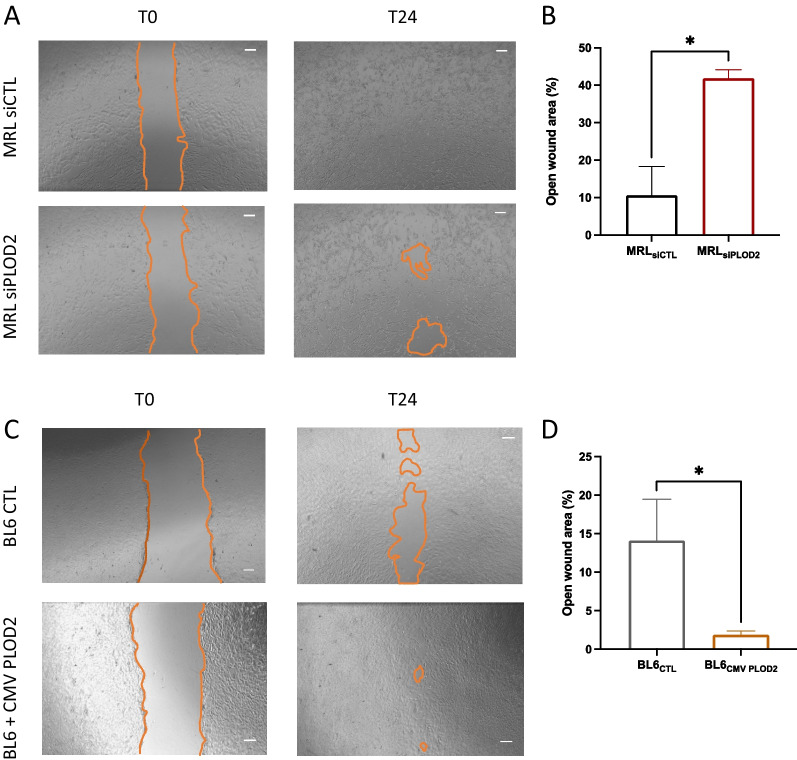
PLOD2 direct MRL MSC migratory ability. **A** Workflow. **B** Representative images of MSC MRL_siCTL_ and MSC MRL_siPLOD2_ and **D** BL6 MSC and BL6 MSC_+CM PLOD2_ scratch assay. The images were taken immediately after the scratches had been made and then after 24 h. The orange line indicates the initiatory and final areas without migrating cells. **C** and** E** Quantitative analysis of the open wound area was performed at 0 and 24 h after wounding using Image J software. 100% corresponds to the highest wound area measured at 0 h. Error bars represent mean ± SEM. *P < 0.05; **P < 0.01; ***P < 0.001, Mann–Whitney unpaired *t* test, two-tailed (*n* = 4–5)

Cell migration has been suggested to be involved in tissue regeneration [39] and recently, we have shown that MRL MSC exhibit a significantly higher migration potential than BL6 MSC [32]. To specifically study the role of *Plod2* on MRL MSC migratory potential in vitro, we analyzed in a scratch wound assay the non-directional migration of MRL and MRL siPLOD2 MSC by evaluating the area of the wound at 24 h post-wounding using Image J software (National Institutes of Health, Bethesda, MD, USA) (Fig. 3A). Representative images from scratch wound healing assay, 24 h post-wounding, indicated an altered resurfacing of the wound for MRL siPLOD2 MSC as compared to MRL MSC (Fig. 3B). The percentage of the open wound area at 24 h which reflects the migration potential of the cells confirmed that MRL siPLOD2 MSC closed the wound significantly slower than MRL MSC (Fig. 3C). Conversely, *Plod2* overexpression in BL6 MSC significantly increased the migration potential of BL6 MSC (Fig. 3D) and decreased the open wound area (Fig. 3E). Altogether these results indicate that *Plod2* expression plays a positive role on the migration potential MSC.

### *Plod2* is necessary for MRL MSC chondroprotective properties

**Fig. 4:**
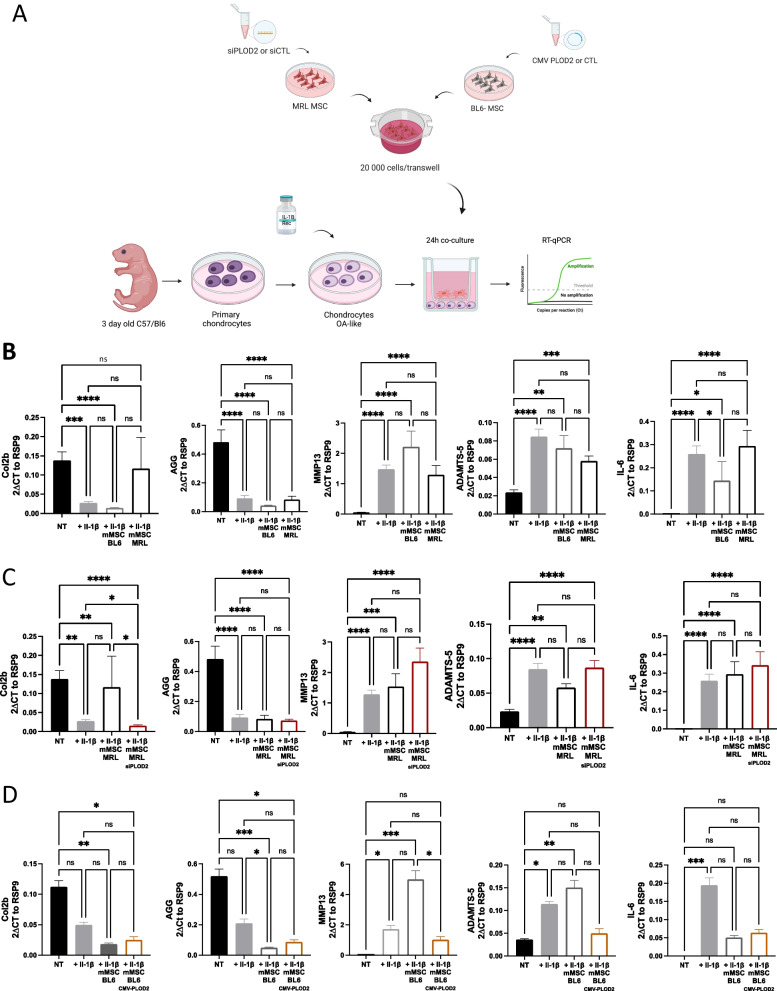
Effect of PLOD2 on chondrocyte gene expression on OA-like chondrocytes. **A** Workflow for the generation of OA-like chondrocytes by incubation with IL1β and their co-culture. **B** RT-qPCR analysis of different chondrocyte and inflammatory markers in control (NT) and OA-like chondrocytes (IL1β) co-cultured or not with MRL MSC and BL6 MSC (*n* = 19). **C** RT-qPCR analysis of different chondrocyte and inflammatory markers in control (NT) and OA-like chondrocytes (IL1β) co-cultured or not with MRL MSC_siCTL_ and MSC MRL_siPLOD2_ (*n* = 19).** D** RT-qPCR analysis of different chondrocyte and inflammatory markers in control (NT) and OA-like chondrocytes (IL1β) co-cultured or not with MSC BL6 and BL6 MSC_+CM PLOD2_ (*n* = 6). Error bars represent mean ± SEM. One-way paired ANOVA, followed by Friedman test for multiple comparison test was performed. ns: 0.1234; *: *P* = 0.332; **: *P* = 0.0021; ***: *P*:0;002 or ****: *P* < 0.0001

MSC protect chondrocytes from the loss of their mature and functional phenotype in vitro and in vivo [27, 31, 40, 41]. Recently, we have shown that *pycr1,* pivotal for MRL MSC glycolysis, contributed to their pro-anabolic function on chondrocytes [42]. We then wondered whether *plod2* highly produced by MRL MSC could protect chondrocytes from a loss of anabolic markers in vitro. To that end, we relied on co-culture experiments with MSC and IL-1β-induced chondrocytes in which chondrocytes exhibit a loss of their anabolic markers including *Col2B* and *Acan* and an increase in their catabolic markers and compared the chondroprotective potential of naïve or genetically modified MRL and BL6 MSC (Fig. 4A). First, we tested the effect of *plod2* silencing on MRL MSC chondroprotective effects on the IL-1β-induced chondrocyte model. While co-culture of IL-1β-treated chondrocytes with MRL MSC transfected with a siCTL (mMSC MRL) tend to protect the chondrocytes from a loss of *Col2B* (Fig. 4B), an anabolic marker, MRL MSC silenced for *plod2* (mMSC MRL_siPLOD2_) did not (Fig. 4C). Moreover, while co-culture of IL-1β-treated chondrocytes with MRL MSC transfected with a siCTL (MRL MSC) tend to protect the chondrocytes from an increase in *Adamts5*, a catabolic marker, MRL MSC silenced for *plod2* (mMSC MRL_siPLOD2_) did not (Fig. 4C). Conversely, *plod2* overexpression in BL6 MSC (mMSC BL6_CMV PLOD2_) significantly protected the chondrocytes from an *Adamts5* increase (Fig. 4D). Altogether, these data show that *plod2* expression by MSC tend to protect chondrocytes from the loss of mature chondrocyte phenotype and the increased expression of catabolic markers which are characteristics of OA.

### MRL MSC protection against OA is mediated by *plod2*

**Fig. 5:**
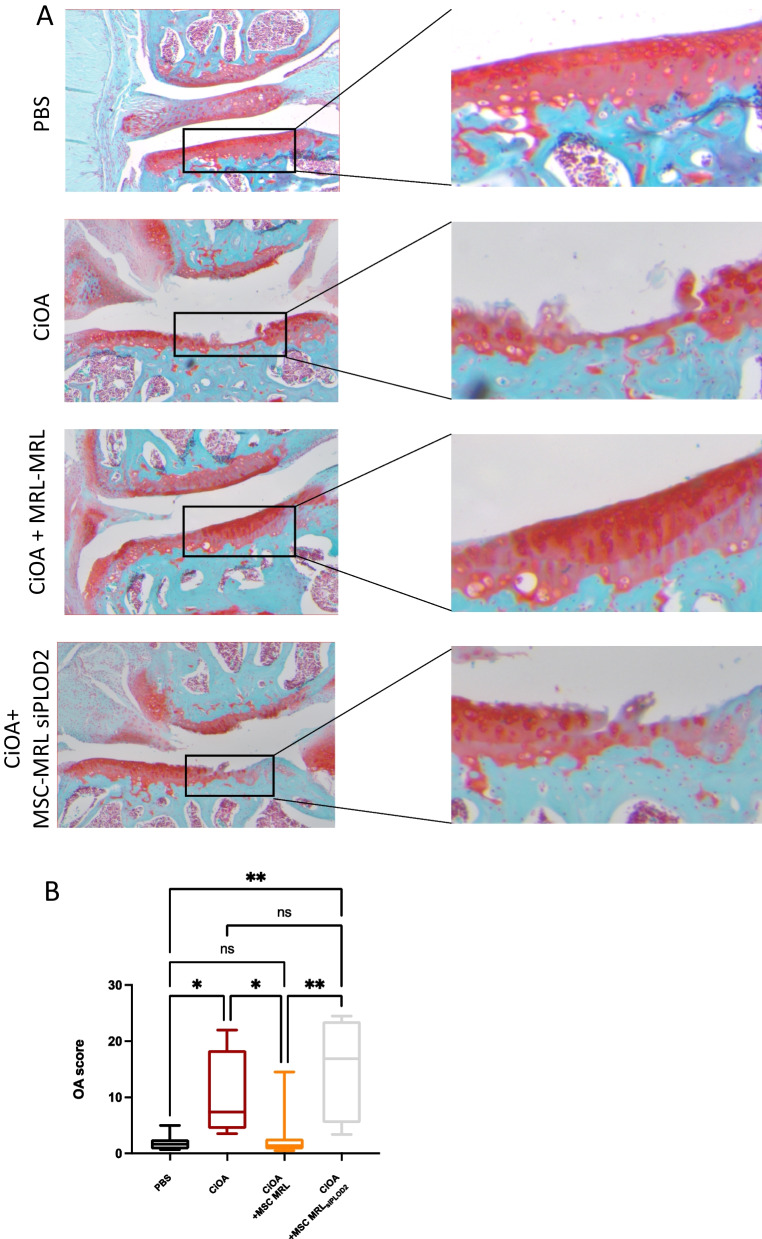
*Plod2* mediates MRL MSC chondroprotective effect from osteoarthritis. **A** Histological sections of CiOA mice not treated (PBS), treated with collagenase only (CiOA), collagenase, MRL MSC (CiOA + MSC MR) and MRL MSC With siRNA for PLOD2 (CiOA + MSC MRL_siPLOD2_) **(B)**. OA score of histological sections of knee joints of the mice described in **(A)** Error bars represent mean ± SEM. One-way ANOVA, (Kruskal–Wallis test) was performed. ns: 0.1234; *: *P* = 0.332; **: *P* = 0.0021; ***: *P*: 0;002 or ****: *P* < 0.0001. *n* = 8 for PBS, CiOA an CiOA + MSC MRL_siPLOD2_and *n* = 9 for CiOA + MSC MRL. Scale bars, 200 µm

We subsequently investigated the role of *plod2* on MRL MSC chondroprotective ability in vivo. For this purpose, we used the collagenase-induced osteoarthritis (CiOA) model, in which mice show signs of cartilage degradation, to test the effect of intra-articular injection of MRL MSC transfected with siCTL (MSC MRL) or siPLOD2 (MSC MRL_siPLOD2_). At D42, histological examination revealed a lower osteoarthritic score in collagenase-treated mice injected with MRL MSC than in collagenase-treated mice without MRL MSC, indicating a protective effect of the MRL MSC (Fig. 5A and B). In contrast, the OA score of mice injected with MRL MSC silenced for *plod2* (MSC MRL_siPLOD2_) was significantly higher than that of untreated mice (injected with PBS) or CiOA mice treated with control MRL MSC (Fig. 5A and B). Those results suggest that *plod2* contributes to the protective effect of MRL MSC against OA.

## Discussion

Unlike most mammals, the “super healer” MRL mouse retains its regenerative capacity in adulthood. Indeed, this mouse can regenerate, among other tissues, its muscles [43], its nervous system [44], and its cartilage [11]. This particularity could explain why this animal model does not develop specific degenerative pathologies such as osteoarthritis or osteoporosis when induced experimentally. To better understand their resistance to joint degenerative diseases, we focused our attention on the secretome of their MSC in comparison with BL6 MSC and demonstrated a specific secretome of MRL MSC with a significantly higher production of PLOD2 compared to BL6 MSC. *Plod2* produced by MRL MSC participate in their specific metabolic status as well as their chondroprotective properties.

PLOD2, involved in lysyl hydroxylation of collagen molecule pivotal for the stability of collagen cross-links [45], is regulated by hypoxia-inducible factor (HIF-1α) [46, 47]. PLOD2 has been intensively studied in tumorigenesis since it promotes aerobic glycolysis in cancer cells [48–50]; our study provides the first evidence for the role of PLOD2 on MRL MSC metabolism, migration potential and chondroprotective properties. Hif-1α plays a crucial role in MSC functions [51] and several if not all mammalian regeneration processes [34]. In MRL mice, after tissue wounding, the biphasic expression profile of *Hif-1α* characterized by a rapid increase in systemic *Hif-1α* levels peaking between days 10 and 14 and decreasing during the second of the regeneration process, suggested the critical role of *Hif-1α* during the process [34, 52]. This was confirmed in experiments showing that *Hif-1α* silencing in MRL mice inhibited ear hole closure and that the injection of drugs stabilizing *Hif-1α* in a hydrogel both proximal and distal to the injured sites led to an accelerated ear hole closure [34, 53]. Since *Hif-1α* is a regulator of *Plod2* expression [46, 47], we assessed the expression level of *Hif-1*α in MRL MSC silenced for *plod2* and found that *Hif-1α* was reduced in MRL MSC transfected with the siRNA against *plod2* as compared to MRL MSC transfected with the siCTL (Additional file 1: Fig. S3A and S3B). Additionally, *Plod2* overexpression in BL6 MSC causes a metabolic modification to reduce oxidative phosphorylation. Altogether, these results suggest that in MRL MSC PLOD2 regulates *hif-1*α expression and that the PLOD2- HIF1α axis controls MRL MSC glycolysis and regenerative properties.

In vitro, in gain and loss of function experiments, we have shown that *plod2* expression is involved in the migration potential of MRL MSC. These results are in line with a study showing that the inhibition of hypoxia-induced PLOD2 reduces the migration and the invasion of glioma cell both in vitro and in vivo [54]. This was associated with an elevated expression of E-cadherin and reduced expression of *vimentin*, *N-cadherin*, *snail* and *slug* in response to PLOD2 suppression. Further experiments should be performed to determine whether the decreased migration potential of MRL MSC in response to *plod2* silencing is due to a modulation of adhesion molecule expression levels.

The silencing of *plod2* in MRL MSC also altered their chondroprotective properties on IL-1β-treated chondrocytes. Indeed, we demonstrated that while MRL MSC protects IL-1β-treated chondrocytes from a loss *Col2B*, MRL MSC silenced *plod2* did not. These results are consistent with our results in the CiOA model where MRL MSC downregulated for *plod2* lose their chondroprotective effect. Therefore, we propose that the chondroprotective effect of MRL MSC relies, in part, on *Plod2* overexpression.

Our results are in contradiction with Bank et al. study suggesting that PLOD2 inhibition and therefore the prevention of the formation of pyridinoline cross-links which stabilize the collagen might favor cartilage repair attempts. They argue that by showing cartilage with collagen-containing low levels of hydroxylysine and pyridinoline might be less prone to degradation induced mechanically [55]. Therefore, the positive role of *Plod2* expression on MRL MSC chondroprotective effect might be due to other properties of *plod2* than that to form collagen cross-links. Stegen and colleagues recently showed that collagen synthesis in chondrocytes was metabolically controlled by *hif-1a* (an inducer of *plod2*) [56]. However, sustained expression of *Plod2* can lead to bone dysplasia, suggesting its involvement in fibrosis. Interestingly, overexpression of PLOD2 via the TGF-B1 pathway in adipose tissue-derived MSC increases the therapeutic potential of MSC in an experimental model of spinal cord injury [57]. In mice with a dominant-negative mutation of the TGF‐β type II receptor, disorganization of collagen fibers was observed [58]. In view of the discrepancy in these results, it would be interesting to know whether the TGF-B1 pathway is also used by the MRL mouse for PLOD2 induction and whether this is beneficial against OA.

We have recently shown that the chondroprotective effect of MRL MSC has been associated with their glycolysis [42]. Indeed, we showed that Pyrroline-5-Carboxylate Reductase 1 *(Pycr1)* downregulation induced MRL MSC metabolism reprogramming specifically associated with a reduced lactate concentration in the extracellular media of the cells. This OXPHOS metabolic reprogramming of MRL MSC knockdown for *Pycr1* induced a loss of MRL MSC chondroprotective functions.

## Conclusions

In conclusion, our findings demonstrate for the first time that the enhanced chondroprotective potential of MRL MSC is attributed, in part, to *Plod2*, which participate in their metabolic specificity, compared with BL6 MSC.

### Supplementary Information

**Additional file 1**. **Fig. S1**. siRNA and plasmid transfection control. **(A)** Full-length Western blot analysis of PLOD2 and Actin, in whole-cell extracts from BL6_CTL_ MSC, BL6 + PLOD2_CMV_ MSC and MRL_siCTL_ MSC, MRL_siPLOD2_ MSC. (B) Analysis of the intensity value of each target protein band was normalized against the intensity value of Actin gel band used as the internal loading control for each sample. **(C)** RT-qPCR analysis of *Plod2* in MRL MSC transfected with control (siCTL) or anti-PLOD2 siRNA (siPLOD2) 24 h post-transfection (n = 3) **(D)** Cell viability of MSC MRL and MSC MRL_siCTL_ measured using CellTiter-glo at 0, 24, 48 and 72 h. (**E)** RT-qPCR analysis of *Plod2* in BL6 MSC transfected with plasmid CMV PLOD2-mCherry (BL6 + CMV PLOD2). **(F)** BL6 MSC transfection with CMV PLOD2 was assessed for mCherry expression. **Fig. S2**. Analysis of OCR and ECAR. Analysis of OCR and ECAR was performed using Seahorse XF analyzer to assess mitochondrial respiration and glycolysis. (**A** and** B**) OCR/ECAR ratio was compared between BL6 MSC and MRL MSC, (**C** and** D**) between MRL MSC_siCTL_ and MRL MSC_siPLOD2_, (**E** and** F**) between BL6 MSC and BL6 MSC_+cmv PLOD2._ OCR and ECAR data were used from Fig. 2. (**A**,**C** and** E**) represent general OCR/ECAR profile, upon successive inhibitor injections. (**B**,**D** and** F**) illustrate OCR/ECAR calculated from baseline phase. (**G**, **H** and **I**) show L-Lactate quantification measured by Elisa Test from culture media harvested after 24 h of culture. Error bars represent mean ± SEM. *P < 0.05; **P < 0.01; ***P < 0.001, Mann–Whitney unpaired t-test, two-tailed. **Fig. S3**. *Hif -1a* mRNA levels in MRL MSC. **(A)** RT-qPCR analysis of *Hif-1a* in MRL MSC transfected with control (siCTL) or anti-PLOD2 siRNAs (siPLOD2) 24 h post-transfection (n = 1) **(B)** RT-qPCR analysis of *Hif-1a* in BL6 MSC and MRL MSC Error bars represent mean ± SEM. *P < 0.05; **P < 0.01; ***P < 0.001, Mann–Whitney unpaired t-test, two-tailed (n = 3).

## Data Availability

The datasets used and/or analyzed in the current study to document the conclusions are provided in the article and in the corresponding supplementary files. They can be made available by the corresponding author on appropriate demand.

## Abbreviation

CiOA: Collagenase-induced osteoarthritis
MRL: Murphy Roths Large
MSC: Mesenchymal stromal/stem cells
ECAR: Extracellular acidification rate
HIF-1a: Hypoxia-inducible factor 1-alpha
OA: Osteoarthritis
OCR: Oxygen consumption rate
PLOD2: Procollagen-lysine,2-oxoglutarate 5-dioxygenase 2
RT-PCR: Reverse transcription-polymerase chain reaction
SEM: Standard Error of the Mean
ADAMTS-5: A disintegrin and metalloproteinase with thrombospondin motifs 5
AGN: Aggrecan
TGF-B: Transforming Growth Factor 1
RSP9: Ribosomal protein S9
ACTB: Actin B
COL2B: Collagen type II B
IL-6: Interleukin 6
