# Supplementary material for: PLOD2, a key factor for MRL MSC metabolism and chondroprotective properties
Source: Stem Cell Res Ther. 2024 Mar 7;15:70. doi: 10.1186/s13287-024-03650-2 (PMC10921602; doi:10.1186/s13287-024-03650-2)
Supplement: Supplementary file 1 — Additional file 1. Fig. S1. siRNA and plasmid transfection control. (A) Full-length Western blot analysis of PLOD2 and Actin, in whole-cell extracts from BL6CTL MSC, BL6 + PLOD2CMV MSC and MRLsiCTL MSC, MRLsiPLOD2 MSC. (B) Analysis of the intensity value of each target protein band was normalized against the intensity value of Actin gel band used as the internal loading control for each sample. (C) RT-qPCR analysis of Plod2 in MRL MSC transfected with control (siCTL) or anti-PLOD2 siRNA (siPLOD2) 24 h post-transfection (n = 3) (D) Cell viability of MSC MRL and MSC MRLsiCTL measured using CellTiter-glo at 0, 24, 48 and 72 h. (E) RT-qPCR analysis of Plod2 in BL6 MSC transfected with plasmid CMV PLOD2-mCherry (BL6 + CMV PLOD2). (F) BL6 MSC transfection with CMV PLOD2 was assessed for mCherry expression. Fig. S2. Analysis of OCR and ECAR. Analysis of OCR and ECAR was performed using Seahorse XF analyzer to assess mitochondrial respiration and glycolysis. (A and B) OCR/ECAR ratio was compared between BL6 MSC and MRL MSC, (C and D) between MRL MSCsiCTL and MRL MSCsiPLOD2, (E and F) between BL6 MSC and BL6 MSC+cmv PLOD2. OCR and ECAR data were used from Fig. 2. (A,C and E) represent general OCR/ECAR profile, upon successive inhibitor injections. (B,D and F) illustrate OCR/ECAR calculated from baseline phase. (G, H and I) show L-Lactate quantification measured by Elisa Test from culture media harvested after 24 h of culture. Error bars represent mean ± SEM. *P < 0.05; **P < 0.01; ***P < 0.001, Mann–Whitney unpaired t-test, two-tailed. Fig. S3. Hif -1a mRNA levels in MRL MSC. (A) RT-qPCR analysis of Hif-1a in MRL MSC transfected with control (siCTL) or anti-PLOD2 siRNAs (siPLOD2) 24 h post-transfection (n = 1) (B) RT-qPCR analysis of Hif-1a in BL6 MSC and MRL MSC Error bars represent mean ± SEM. *P < 0.05; **P < 0.01; ***P < 0.001, Mann–Whitney unpaired t-test, two-tailed (n = 3). [file 13287_2024_3650_MOESM1_ESM.pptx]

## Slide 1
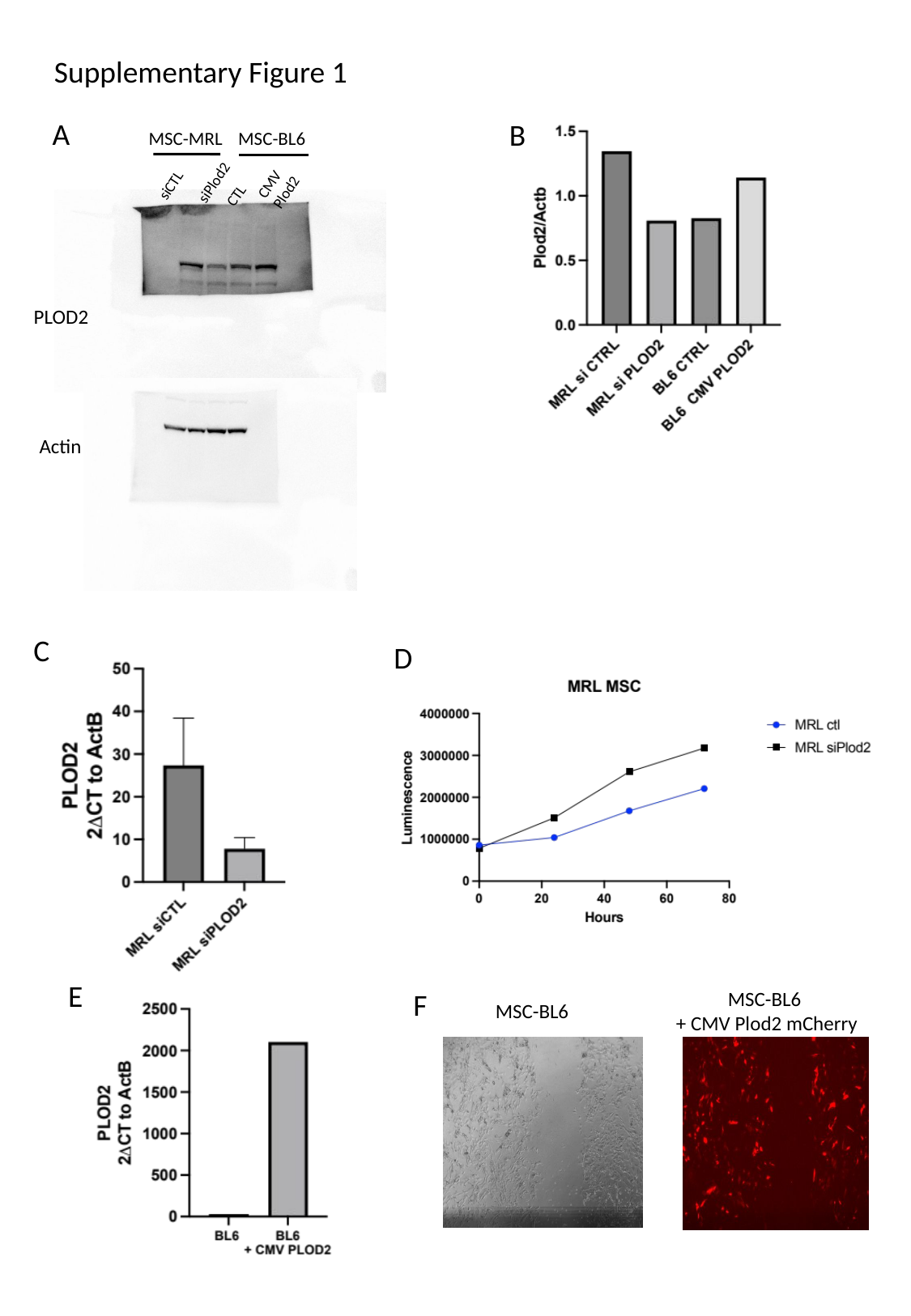

Supplementary Figure 1
A
B
MSC-MRL
MSC-BL6
CMV
Plod2
siPlod2
siCTL
CTL
PLOD2
Actin
C
D
E
F
MSC-BL6
+ CMV Plod2 mCherry
MSC-BL6

## Slide 2
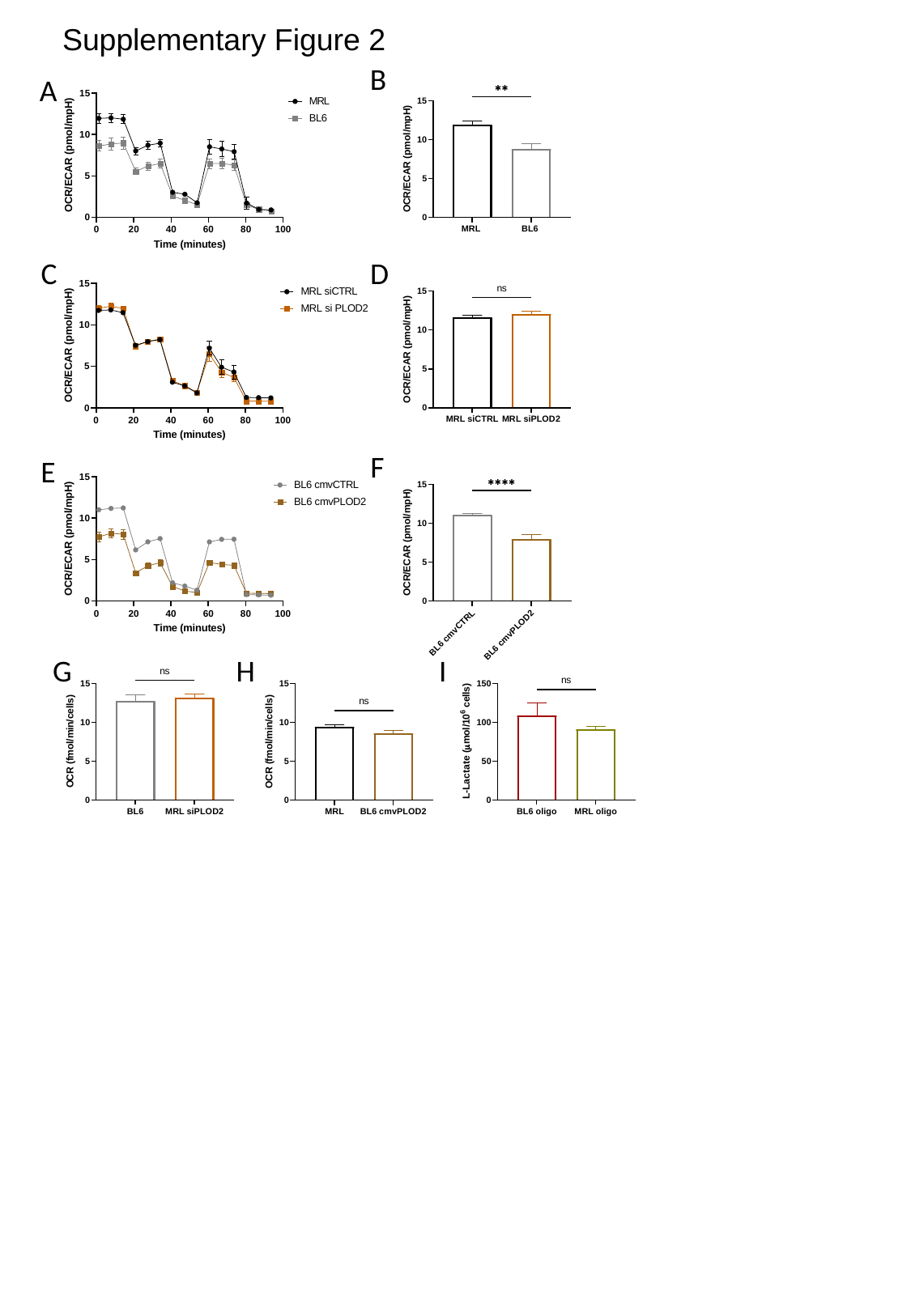

Supplementary Figure 2
B
A
C
D
F
E
G
H
I

## Slide 3
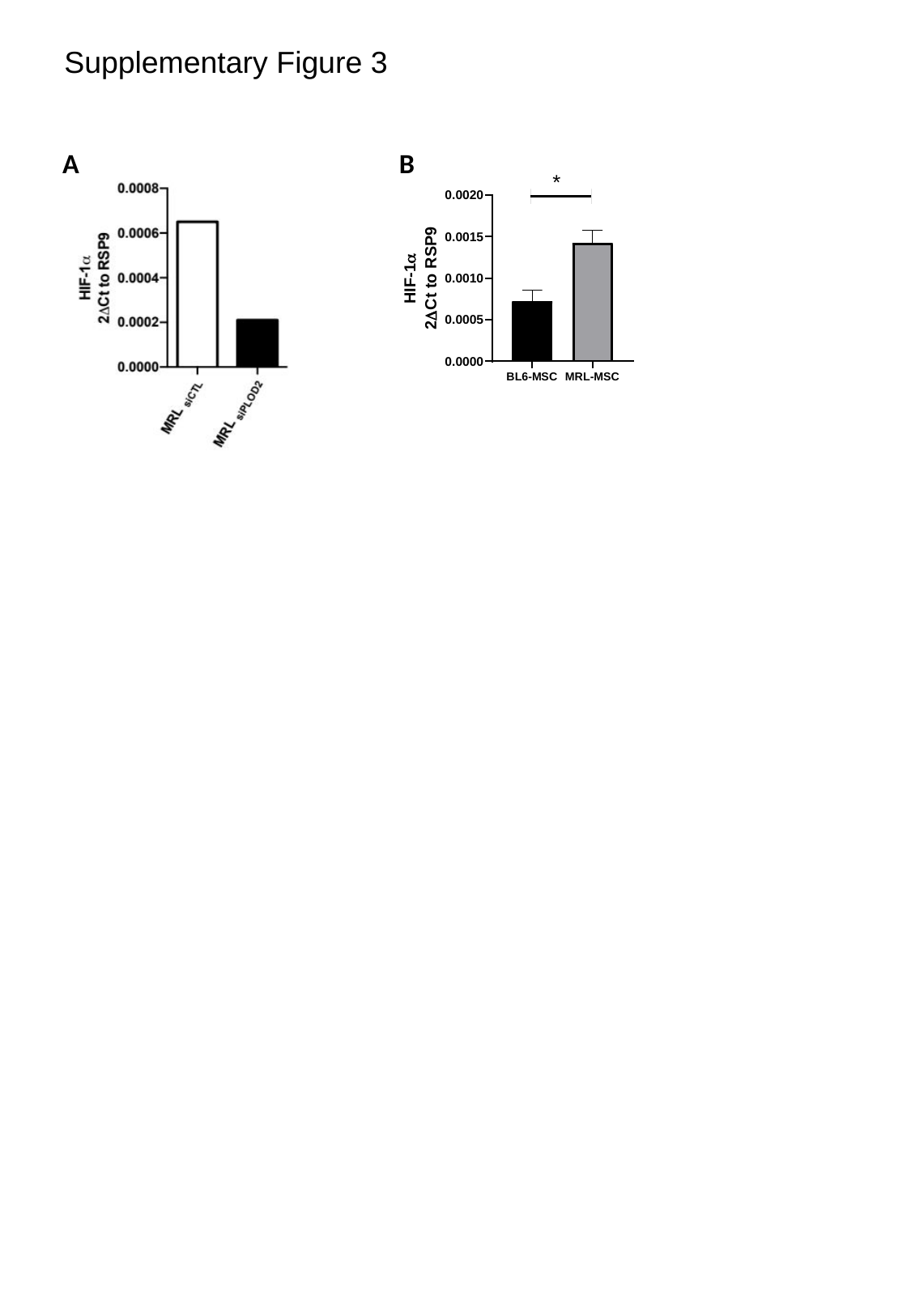

Supplementary Figure 3
A
B
